# Change in quality of life and return to work and sports after isolated closing-wedge distal femoral osteotomy

**DOI:** 10.1186/s13102-023-00721-4

**Published:** 2023-10-10

**Authors:** Qian Fang, Dong Wang, Wenzheng Liu, Wei Lin, Guanglin Wang

**Affiliations:** 1 Trauma Medical Center, Department of Orthopaedic Surgery, West China Hospital of Sichuan University, Chengdu, China; 2 West China Women’s and Children’s Hospital of Sichuan University, Chengdu, China

**Keywords:** Closing-wedge distal femoral osteotomy (CWDFO), Health-related quality of life (HRQoL), Return to work, Return to sports

## Abstract

**Purpose:**

To analyze return to work and sports, and health-related quality of life (HRQoL) after closing-wedge distal femoral osteotomy (CWDFO) for valgus deformity and lateral compartmental osteoarthritis.

**Methods:**

Thirty-three patients underwent isolated CWDFO in our center between January 2018 and June 2020 were enrolled, of whom 32 and 23 patients were included in the return-to-work and return-to-sports analyses, respectively. Short Form-36 (SF-36), Tegner score, Knee injury and Osteoarthritis Outcome Score (KOOS) and visual analog scale (VAS) pain score were compared preoperatively and postoperatively. And postoperative complications were recorded.

**Results:**

Overall, 33 patients were contacted at a mean follow-up of 37.94 ± 6.68 months, with a median age of 35 years (range: 26–63 years) at the surgery time. The physical component summary of SF-36 (*p* < 0.001) increased significantly at 1 year postoperatively. All patients returned to work, including 96.86% who returned to the same level of work in 1.89 ± 0.98 months, and to sports, including 78.26% who returned to the same sport level in 6.50 ± 2.05 months. Rates of returning to work (*p* = 0.215) and sports (*p* = 0.165) did not differ with work/sports intensity. Tegner scores (*p* = 0.025) and VAS pain scores (*p* < 0.001) decreased, and KOOS (*p* < 0.001) increased at 1 year postoperatively. Revision/conversion surgery was not required. In all, 30.43% patients reported a subjective decrease in sports ability; 82.61% patients considered their sports ability acceptable.

**Conclusion:**

Patients returned to work/sports after isolated CWDFO, and had increased HRQoL. Patients playing high-impact sports had lower rates of returning to the same sport level, and may require preoperative counseling.

**Level of evidence:**

IV, Case series.

## Introduction

Valgus deformity of the lower limb can lead to decreased contact area, increased contact pressure and changed knee kinematics in the lateral compartment of the knee, as detected by biomechanical tests and computational simulation in silico [1–3], which results in early progression to osteoarthritis and impairs the patient’s quality of life and ability to participate in work and sports [4]. Total knee arthroplasty (TKA) and unilateral knee arthroplasty are effective choices for treating osteoarthritis in older patients [5]. However, for young patients, the risk of failure of arthroplasty and requirement of revision surgery are higher, and the patient-reported outcomes are worse [6, 7]. Performing an osteotomy around the knee to correct the valgus deformity is an effective alternative treatment for young patients who have a higher activity demand [8, 9]. Isolated closing-wedge distal femoral osteotomy (CWDFO) is one of the knee-preservation surgeries used to correct the mechanical axis of the lower limb [10, 11], contributing decrease of the contact pressure, increase of the contact area and accomplishment of normal knee kinematics in the lateral compartment of the knee [12, 13]. CWDFO has been proven to correct the mechanical axis of the lower limb and slow down the progression of osteoarthritis in long-term follow-up studies [14, 15]. The ability to return to work and sports is crucial for active and young patients, and keeping away from work and sports might cause social and psychological stress to such patients [16]. Although the health-related quality of life (HRQoL) and rates of return to work and sports have been reported for patients treated with high tibial osteotomy (HTO), opening-wedge distal femoral osteotomy (OWDFO), and double-level osteotomy (DLO) [17–19], few studies have reported these parameters after CWDFO. Therefore, this study aimed to assess the ability and timeline of patients’ return to work and sports as well as evaluate the changes in their quality of life after isolated CWDFO for the treatment of osteoarthritis. We hypothesized patients could have satisfactory rate of return to work and sports with increased quality of life postoperatively.

## Methods

### Patient enrollment and ethical approval

Ethical approval of this study was obtained from our institutional review board. We retrospectively collected the chart records of patients who underwent isolated CWDFO in our center between January 2018 and June 2020. Patients with previous surgeries on the lower limbs, other surgeries on the lower limb during follow-up, or incomplete follow-up data were excluded.

### Surgical techniques and rehabilitation

**Fig. 1:**
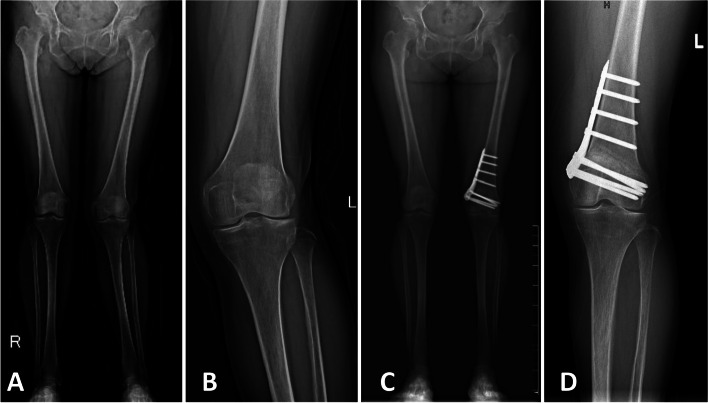
Preoperative (**A**) and postoperative (**C**) full-length weight-bearing radiographs of the lower limbs, and preoperative (**B**) and postoperative (**D**) anterior–posterior view radiographs of the affected knee of patients with isolated valgus deformity and lateral compartmental osteoarthritis

Surgeries were planned based on full-length weight-bearing radiographs of the lower limbs. The indications for the surgery were valgus malalignment of more than 5° and lateral compartment osteoarthritis. The weight-bearing axis was targeted to be corrected at a mechanical axis percentage of 45%. The mechanical axis percentage was defined as the horizontal distance from the medial edge of the tibial plateau to the weight-bearing axis on tibial plateau divided by the width of the tibial plateau, with the medial edge as 0% and the lateral edge as 100%. The osteotomy wedge was created under the guidance of Kirschner wires, and was removed with the lateral cortex intact. The osteotomy site was closed using a continuous gentle varus-directed force with stabilization of the knee. The osteotomy site was fixed with a Tomofix plate (Tomofix™ medial orthopedic plate of distal femoral osteotomy, DePuy Synthes, 04.120.550S and 04.120.551S), and fluoroscopy was used to confirm the correction. The remaining distal holes were filled with screws after final confirmation of the correction. Representative preoperative and postoperative radiographs are shown in Fig. 1.

Patients were restricted to partial weight-bearing for 6 weeks after the surgery, and then, progressed to full weight-bearing depending on radiographic progression. No braces or casts were applied and the full range of movement of the knee was allowed immediately after the surgery. And exercises including heel raises, eccentric quadriceps and hamstring strengthening and gait normalization were progressively advanced. All patients were followed up for at least 1 year postoperatively in the outpatient clinic, and further follow-up was conducted via telephone calls. We recorded postoperative complications such as fracture around the osteotomy site, non-union or delayed union of the osteotomy site, wound healing, infection, loss of correction, thromboembolic events, and subjective reports of pain and stiffness around the osteotomy site and so on.

### Outcome measurement

The patients’ HRQoL was measured using the Short-Form 36 (SF-36 V2) Health Survey. This form includes the following 8 subscales: physical functioning (PF), role limitations due to physical health (RP), bodily pain (BP), and general health (GH) as physical components; vitality (VT), social functioning (SF), role limitations due to emotional problems (RE), and mental health (MH) as mental components. The physical component summary (PCS) was calculated by summing the PF, RP, BP, and GH scores, and the mental component summary (MCS) was calculated by summing the VT, SF, RE, and MH scores. We compared the preoperative and postoperative scores as well as compared the patients’ scores with those of the general population (GP), for which, the parameter value was set as 50.

Patients who had worked within 1 year preoperatively were included in the return-to-work analysis. To evaluate the change in workload after the surgery, the patients’ jobs were classified into 4 categories according to their physical demands: sedentary, low, moderate, and heavy work. This classification has been used in multiple orthopedic studies [20, 21]. The timeline and rate of return to work after the surgery were recorded.

Patients who participated in sports within 1 year preoperatively were included in the return-to-sports analysis. The sports that the patients engaged in were classified as low-, moderate-, and high-impact sports, according to a previous report [22]. The timeline of the patients’ return to sports was also noted. In addition, patients were asked to subjectively evaluate their postoperative sports ability as unchanged or decreased compared to the preoperative level. The Tegner activity scale was also used for the evaluation of sports participation.

In addition, the visual analog scale (VAS) pain score and Knee injury and Osteoarthritis Outcome Score (KOOS) were recorded preoperatively and at 1 year postoperatively.

### Statistical analysis

Statistical analyses were performed using SPSS *v*26 (IBM Corp., Armonk, NY, USA). We compared the preoperative and postoperative values of the included variables. The Kolmogorov–Smirnov test was used to assess the normality of the distribution of continuous variables. Normally distributed continuous variables were reported as mean and standard deviation, and were compared using the one-sample, independent, or paired *t*-test. Non-normally distributed continuous variables were reported as median and interquartile range, and were compared using the one-sample, independent (Mann–Whitney U test), or paired nonparametric test (Wilcoxon signed-rank test). Categorical variables were reported as number and percentage, and were compared using the chi-square test or Fisher exact test. Intra- and inter-observer reliability were evaluated using intraclass correlation coefficients (1 = highly reliable; 0 = unreliable) by two blinded clinicians with more than 5 years of working experience in orthopedics. A two-tailed *p*-value of < 0.05 was considered significant.

## Results

### Clinical information

**Fig. 2:**
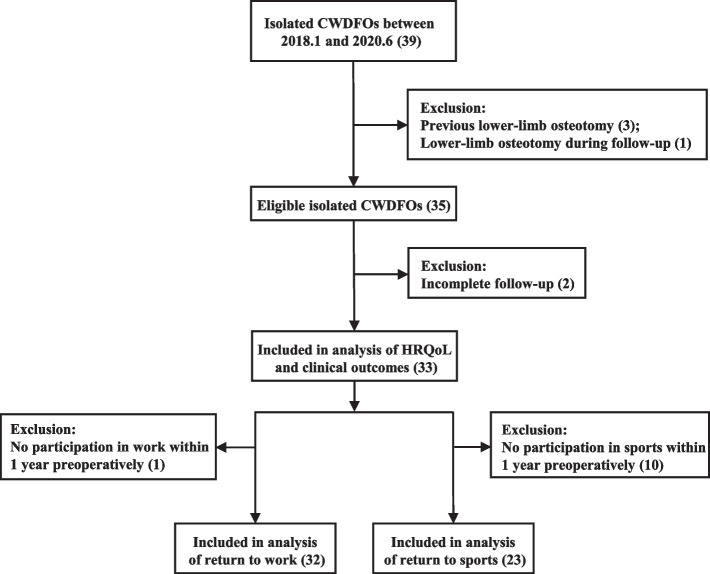
Flow chart of inclusion and exclusion

A total of 39 patients underwent isolated CWDFO in our hospital during the study period. We excluded 3 patients with previous lower-limb osteotomy, 1 patient with H, and 2 patients with incomplete follow-up. Thus, finally, 33 patients with lateral-compartment osteoarthritis were included in this study (Fig. 2). Their mean follow-up duration was 37.94 ± 6.68 months. The demographic data of the included patients are presented in Table 1. The intra- and inter-observer reliability were both higher than 0.8 for the correction angle, and were 0.537 (0.343, 0.688) and 0.598 (0.419, 0.732), respectively, for the Kellgren-Lawrence grade.

**Table 1:** Demographic data of the study patients

Number of patients	33
Mean age (years)	35 (26–63)
Gender (male/female)	4/29
Body mass index (kg/m^2^)	21.26 ± 1.96
Affected side (right/left)	15/18
Follow-up duration (months)	37.94 ± 6.68
Kellgren-Lawrence grade	1.55 ± 0.47
Correction angle (degree)	8.36 ± 1.60

### Health-related quality of life

All SF-36 subscale scores and the PCS were significantly increased at 1 year postoperatively, as compared to the preoperative values. No significant difference was found in the pre-and postoperative MCS (*p* = 0.090). Compared with the GP score, the preoperative MCS was significantly higher; the MH score showed no significant difference (*p* = 0.794), and the other subscale scores and the PCS were significantly lower. At 1 year postoperatively, the PCS (*p* = 0.182) and PF score (*p* = 0.346) showed no significant difference from the GP score, while the other subscale scores and the MCS were significantly higher than the GP score. The above results are presented in detail in Table 2.

**Table 2:** Results of the Short Form-36 health survey

Score	Preoperative	Postoperative (1 year)	*p* value	*p* value (GP *vs*. preoperative value)	*p* value (GP *vs*. postoperative value)
PCS	31.03 ± 8.22	50.78 ± 3.28	< 0.001	< 0.001	0.182
PF	34.06 ± 10.53	51.43 (42.52–57.37)	< 0.001	< 0.001	0.346
RP	31.06 (15.99–43.12)	52.16 (46.13–55.17)	< 0.001	< 0.001	0.020
BP	30.23 (18.93–47.18)	52.83 (41.53–58.48)	< 0.001	< 0.001	0.003
GH	48.35 (30.32–61.23)	58.65 (50.92–63.80)	< 0.001	0.039	< 0.001
MCS	53.66 ± 9.97	56.83 ± 3.23	0.090	0.043	< 0.001
VT	45.46 (26.82–56.64)	58.22 (49.19–64.10)	< 0.001	0.008	< 0.001
SF	44.07 (21.42–59.17)	54.13 (44.07–59.17)	< 0.001	0.013	< 0.001
RE	48.88 (25.33–53.59)	53.59 (44.17–58.30)	0.001	0.013	0.023
MH	57.29 (18.33–60.55)	57.30 (47.56–63.79)	0.009	0.794	< 0.001

*GP* General population, *PCS* Physical component summary, *PF* Physical functioning, *RP* Role limitations due to physical health, *BP* Bodily pain, *GH* General health, *VT* Vitality, *SF* Social functioning, *RE* Role limitations due to emotional problems, *MH* Mental health

### Return to work

Of the 33 patients included in the study, 32 patients had worked within 1 year preoperatively, and were included in the return-to-work analysis. All 32 patients returned to work postoperatively, and 96.86% of them returned to their previous level of work. One patient switched from heavy work to moderate work postoperatively, and returned to work at 3.46 months after the surgery. The rates of return to work did not significantly differ with the work intensity (*p* = 0.215). The rate and timing of return to work at the previous work intensity are presented in Table 3.

**Table 3:** Time and rate of return to work and sports

	**Preoperative**	**Postoperative**	***p* value**	**Time (month)**	**Rate**
**Work**				**Return to work**	
Sedentary	3	3	0.988	0.88 ± 0.25	100%
Light	11	11		1.20 ± 0.41	100%
Moderate	12	13		2.17 ± 0.57	100%
Heavy	6	5		3.37 ± 0.86	83.33%
Overall	32	32		1.89 ± 0.98	96.86%
**Sports**				**Return to sports**	
Low impact	8	12	0.599	5.60 ± 1.35	100%
Moderate impact	10	8		6.87 ± 1.93	70%
High impact	5	3		8.45 ± 3.06	60%
Overall	23	23		6.50 ± 2.05	78.26%

### Return to sports

In all, 23 patients had participated in sports within 1 year preoperatively, and were included in the return-to-sports analysis. All 23 patients returned to sports postoperatively, and 78.26% returned to their previous sport level in 6.50 ± 2.05 months after the surgery. Details of the time and number of patients who returned to sports with the same impact intensity as the preoperative level are presented in Table 3. One patient in the high-impact group switched to the moderate-impact group, while one patient in the high-impact group and 3 patients in the moderate-impact group switched to the low-impact group. The rate of return to sports did not significantly differ between sports with varying levels of impact (*p* = 0.165). In the subjective evaluation, 7 patients (30.43%) reported a decreased ability to play sports at the final follow-up, though 4 of these patients considered that their current sports ability was acceptable. Thus, in total, 20 patients (86.96%) thought their postoperative sports ability was acceptable. The Tegner score remained unchanged, from 4.00 (3.00–7.00) prior to symptom onset to 4.00 (3.00–7.00) at 1 year postoperatively (*p* = 0.025). The specific return to sports rates were as follows: volleyball (1 of 2, 50.00%), basketball (1 of 3, 33.33%), soccer (1 of 2, 50.00%), gymnastic training (2 of 3, 66.67%), badminton (1 of 2, 50.00%), running (2 of 4, 50.00%), table tennis (6 of 9, 66.67%), yoga (4 of 4, 100.0%), cycling (14 of 18, 77.78%), square dancing (4 of 4, 100.0%), and swimming (12 of 12, 100%).

### Clinical outcomes and postoperative complications

The pain intensity as measured using the VAS decreased from 4.00 (2.00–7.00) preoperatively to 2.00 (0.00–5.00) at 1 year postoperatively (*p* < 0.001). The KOOS increased from 258.07 ± 42.79 preoperatively to 469.79 ± 23.15 at 1 year postoperatively (*p* < 0.001). The hinge fracture occurred in one patient at 2 weeks postoperatively, but bone healing was achieved at 6 months postoperatively. All patients achieved bone healing by 6 months postoperatively. No cases of complications such as wound healing, infection, loss of correction, and thromboembolic events were reported. No patient required revision surgery or conversion to total knee arthroplasty. In all, 6 patients (26.09%) reported stiffness, 4 patients (17.39%) reported occasional pain, and 3 patients (13.04%) reported chronic pain (Table 4).

**Table 4:** Postoperative complications

Hinge fracture	1 (3.03%)
Osteotomy collapse	0
Metalwork failure	0
Non union	0
Delayed union	0
Wound dehiscence	0
Infection	0
Loss of correction	0
Thromboembolic events	0
Stiffness	6 (18.18%)
Occasional pain	4 (12.12%)
Chronic pain	3 (9.09%)
**Total complications**	**9 (27.27%)**

## Discussion

The present study revealed that 96.86% of patients who underwent isolated CWDFO for the treatment of osteoarthritis could return to the same level of work in 1.89 ± 0.98 months, and 78.26% of them could return to the same level of sports in 6.50 ± 2.05 months. Moreover, the patients’ HRQoL was increased postoperatively and was comparable to that of the GP.

Osteoarthritis subsequent to valgus deformity affects patients’ quality of life by impairing knee function and causing psychological and social stress. This impact can be more prominent in young and active patients because of a higher expectation of return to work and sports. Thus, multifactorial measurement of postoperative outcomes, covering both physical and mental components, is necessary. The HRQoL as measured by the SF-36 is widely used after orthopedic surgeries and for osteoarthritis patients [23, 24]. In the present study, all SF-36 parameters were lower in the patients preoperatively than in the GP, except for the MH score, which reflects the negative effects of lateral osteoarthritis due to valgus deformity. The patients’ PCS and all subscale scores were significantly increased at 1 year postoperatively. Although the mean MCS was also elevated postoperatively, this change was not significant. The above findings indicate a significant benefit of CWDFO both physically and mentally. Similar effects have been reported in previous studies on HTO and DLO [17, 18, 25]. Ihle et al. reported a significant increase in the PCS and all subscale scores, instead of MCS in 24 patients of DLO with follow-up of 18.0 ± 10.0 (5–43) months [17]. Saier et al. reported significant increased PCS and MCS over time in 46 patients of HTO with follow-up of 24 months and it is also suggested improvement of HRQOL reached the peak in 6–12 months postoperatively, and the value remained constant thereafter [18].

It is important to explore the ability and timeline of return to work and sports for young and active patients who undergo isolated CWDFO. Studies have reported the rate and time of return to work and sports after HTO and OWDFO, but these studies focused mainly on the opening-wedge technique [8, 20]. Hoorntje et al. reported the postoperative work ability of 80 patients who underwent distal femoral osteotomy via multiple techniques; 91% patients could return to work, and 81% achieved this within 6 months postoperatively [26]. Puzzitiello et al. reported that 32 patients who had undergone OWDFO returned to work after a median of 3.0 months and a mean of 6.0 ± 13.2 months; moreover, 71% patients returned to work at the same intensity level [21]. Agarwalla et al. reported that 70.6% of patients who underwent OWDFO returned to sports in 9.5 ± 3.3 months, and only half of them returned to the same intensity level of sports [19]. Liu et al. reported that patients treated with isolated opening-wedge HTO returned to sports in 7.5 ± 5.0 months, while 41.2% of them returned to the same intensity level of sport [27]. In our study, all patients could return to work, and 96.86% patients returned to work at the same level as previously after a mean of 1.89 ± 0.98 months; in addition, all patients returned to sports, and 78.26% of them returned to the same sport-intensity level in 6.50 ± 2.05 months. The higher rate and lower time of returning to the same level of work and sports may be attributable to the different technique and unilateral-only operation in this study. Compared with the opening-wedge technique used in previous reports [28, 29], the closing-wedge technique provides correction without an osteotomy gap. This might lead to quicker bone healing and faster recovery of knee function. Jacobi et al. claimed that after OWDFO, bone healing took a long time; sufficient consolidation was detected at 3 months postoperatively in 7 of their patients (50%) and at 6 months postoperatively in 12 patients (86%) [30]. In contrast, Nakashima et al. reported that 80% of patients treated with closing-wedge HTO returned to sports at the same intensity level in 7.2 ± 3.1 months [31]. Our study showed a comparable time and rate of returning to sports. However, direct comparison between the opening-wedge and closing-wedge techniques is needed to accurately analyze the difference in the rates of return to work after these two surgeries.

Patients with a lower preoperative work intensity tended to return to their preoperative level of work in a shorter time. Although patients with a higher work intensity had to wait a longer time to resume work, their ability to eventually return to work was not affected. The rate of return to work did not differ between different work intensities (*p* = 0.215). These findings are comparable to those of previous reports [20, 21]. The postoperative rehabilitation protocol is also thought to influence the time required to return to work [20]. Since most studies advised 6–8 weeks of partial weight-bearing, patients were allowed to return to sedentary or light work during this time, while patients with higher work intensities were advised to return to work after this time. Psychosocial factors have also been reported to be associated with patients’ motivation to return to work [32, 33]. It is suggested that patients without work compensation were more motivated to return to work [34]. Hoorntje et al. reported that patients who were the breadwinners of their families (contributing more than 50% of the family income) and those who lacked preoperative sick leave had higher rates of return to work at 6 months postoperatively [35]. In the current study, all patients were covered by medical insurance and had postoperative paid sick leave; nevertheless, all patients returned to work in 6 months after the surgery. Nine patients were the breadwinners of their families, according to the above definition; no significant difference was detected between breadwinners and non-breadwinners in terms of the time required to return to work at their preoperative level (*p* = 0.697). This difference between our study and previous findings might result from our small sample size, the different insurance statuses of our patients, and the different technique used in our study.

A study has indicated that the preoperative sports level was related to the rate of return to sports [31]; however, this was not detected in the current study (*p* = 0.165). Although the difference was not significant, low-impact sports were associated with a higher rate and lower time of return to sport. High-impact sports (e.g., soccer and basketball) involve more cutting and lateral movements, and have higher physical demands, which limited early participation in them. Thus, preoperative education is needed to manage patients’ expectations about returning to high-impact sports [19].

One review reported that distal femoral osteotomy was associated with a reoperation rate of 25.7% (excluding plate removal) during a follow-up of 3–7.5 years and a rate of conversion to total knee arthroplasty of 4.0% ± 2.9% [8]. No reoperation or conversion was required in our study. The high reoperation rates in previous studies might result from the inclusion of military personnel, who have a higher level of physical activity [36]. Longer follow-up is needed for the detection of a difference in the rate of conversion surgery.

Our study also has several limitations. The small sample size and retrospective study design might cause selection bias. The majority of the included patients were female. Moreover, the follow-up duration was short. The SF-36 and KOOS questionnaires are very long and easily cause respondent fatigue, so we only reported the results at 1 year postoperatively, which was covered by the follow-up in our outpatient clinic.

## Conclusion

Patients who undergo isolated CWDFO can be allowed to return to work and sports. However, return to high-impact sports might be delayed or unlikely, and preoperative education is needed for the management of patients’ expectations.

## Data Availability

The datasets used and/or analyzed during the current study are available from the corresponding author on reasonable request.

## Abbreviations

CWDFO: Closing-wedge distal femoral osteotomy
HRQoL: Health-related quality of life
PCS: Physical component summary
MCS: Mental component summary
HTO: High tibial osteotomy
OWDFO: Opening-wedge distal femoral osteotomy
DLO: Double-level osteotomy
PF: Physical functioning
RP: Role limitations due to physical health
BP: Bodily pain
GH: General health
VT: Vitality
SF: Social functioning
RE: Role limitations due to emotional problems
MH: Mental health
GP: General population
